# High incidence of hyponatremia in trained rowers during a four-week training camp

**DOI:** 10.1186/2052-1847-7-S1-O17

**Published:** 2015-08-11

**Authors:** Gunnar Treff, Constantin Ulrich Mayer, Wiebke Kristin Fenske, Katja Blouin, Jürgen Michael Steinacker, Bruno Allolio

**Affiliations:** 1 Department of Sports and Rehabilitation Medicine, University of Ulm, Ulm, Germany; 2 Department of Internal Medicine I, Endocrine Unit, University Hospital, University of Wuerzburg, Wuerzburg, Germany; 3 Leipzig University Medical Center, IFB Adiposity Diseases, Leipzig, Germany; 4 Comprehensive Heart Failure Center, University of Wuerzburg, Wuerzburg, Germany

## Purpose

The aim of this study was to investigate the incidence of hyponatremia during 28 days of high volume rowing training.

## Methods

30 members of the German junior rowing team (21 males, 9 females) were studied during a training camp of four weeks duration. Serum sodium ([Na^+^]) and osmolality were assessed at baseline before entering the training camp, and at day 7, day 13, day 18, day 24, and day 28. Additionally, daily fluid intake, body mass, urine parameters and training volume were determined.

## Results

Hyponatremia ([Na^+^] < 135 mmol/L) was observed in 70 % of the rowers. The highest incidence amounted to 43% at day 18, when training volume was highest. [Na^+^] decreased from 143 ± 8.7 mmol/L (baseline) to 134.5 ± 5.4 mmol/L (day 18, p< 0.01). Hyponatremia was associated with body mass gain in the preceding 24 hours (p<0.01). [Na^+^] returned to normal values at day 28 (139.8 ± 3.9 mmol/L). Relative fluid intake (L/m^2^ body surface area) increased from day 7 (males: 2.79 ± 0.78 L/m^2^; females: 2.20 ± 0.70 L/m^2^) to day 28 (3.88 ± 0.69 L/m^2^ and 2.65 ± 0.93 L/m^2^; p<0.05). No athlete developed symptomatic hyponatremia.

## Discussion

Prolonged high volume rowing training can lead to a high incidence of hyponatremia. The observed hyponatremia is associated with increases in body mass, which is a main characteristic of ‘exercise induced hyponatremia’. However, other mechanisms are probably also involved in the development of the observed hyponatremia, like e.g. inadequate suppression of antidiuretic hormone secretion. We conclude that overdrinking should be avoided during high volume training camps and adequate Na^+^-availability for athletes should be ensured. If central symptoms occur during training camps, team doctors should take hyponatremia into account.

